# Oxidation and hydrogenation of Pd: suppression of oxidation by prolonged H_2_ exposure

**DOI:** 10.1039/c9ra00436j

**Published:** 2019-03-19

**Authors:** Takehiro Tamaoka, Hideto Yoshida, Seiji Takeda

**Affiliations:** The Institute of Scientific and Industrial Research, Osaka University 8-1 Mihogaoka Ibaraki Osaka 567-0047 Japan h-yoshida@sanken.osaka-u.ac.jp; Department of Materials and Manufacturing Science, Graduate School of Engineering, Osaka University 2-1 Yamadaoka Suita Osaka 565-0871 Japan

## Abstract

We investigate the phase transition of a Pd surface in both oxidizing and reducing environments by environmental transmission electron microscopy (ETEM). ETEM allows us to study sequential exposure of Pd to O_2_ and H_2_ in the same TEM conditions. First, under ETEM observation, oxidation occurs at step edges but it can also occur at terraces. Second, as the most important result, we observed a novel process where previous exposure to H_2_ suppresses new oxidation of the Pd surface. Third, we show by electron energy loss spectroscopy (EELS) that this process, suppression of oxidation by previous exposure to H_2_, is not due to the formation of bulk β-phase Pd hydride. We also demonstrate that this process is not present in Pt. Finally, we discuss the hypothesis to explain this phenomenon: formation of surface–Pd–hydride suppresses the new oxidation. This observation, suppression of oxidation by H_2_ exposure, may eventually lead to new breakthroughs.

## Abstract 2

Oxidation of the Pd surface exhibits dependence on the history of pre-exposure to H_2_.

## Graphical abstract

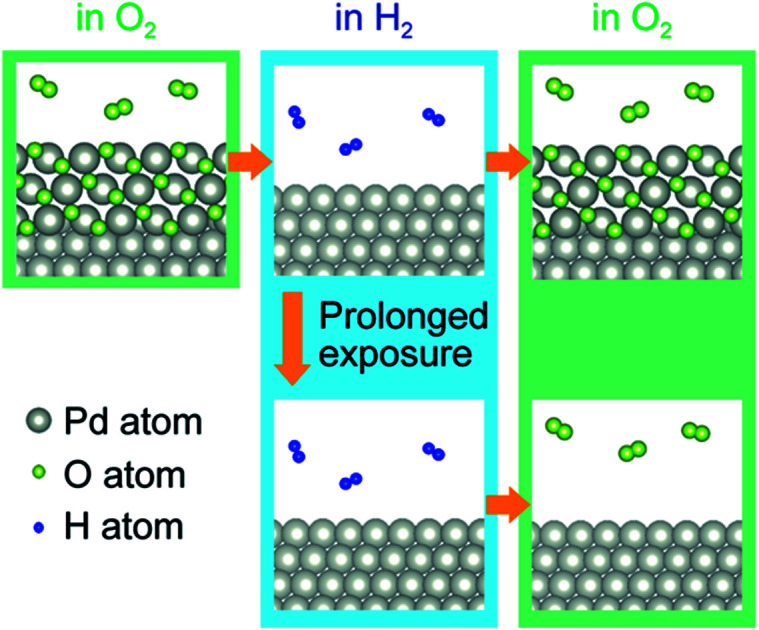

## Introduction

Studying reaction processes at gas–metal interfaces are important in research fields such as catalysis and gas sensing. However, the reaction processes at the atomic scale remain poorly understood because these reactions happen in the atomic scale, they are fast, and they are complex. Additionally, obtaining high resolution images and spectra at reaction environments is difficult. To this day, in order to understand atomic-scale reaction processes at the interface, a lot of methodologies have been developed such as STM,^1,2^ AFM,^3^ EELS,^4,5^ and XPS.^2^ Among these techniques, environmental transmission electron microscopy (ETEM) is a powerful tool because of its high spatial and temporal resolution under a reaction environment. Although ETEM requires high energy electrons that may damage the specimen, this technique has been extremely useful in elucidating many reaction processes.^6–9^

In this study, we focus on the oxidation and reduction of Pd surfaces. The interface between Pd and gases, such as O_2_ and H_2_, is important because Pd is used in various applications such as catalysis,^10–12^ hydrogen sensing,^13,14^ and hydrogen storage.^15,16^ Therefore, there have been studies about adsorbed states of the gas molecules,^1,17^ surface reconstructions,^18,19^ and surface phase transitions.^20^ These results have been useful, but the research field can clearly benefit from dynamic information, and this opens opportunities for ETEM studies. Zhang *et al.* studied individual Pd nanoparticles under O_2_ and suggested that the oxidation starts from step edges.^21^ Baldi *et al.* combined E(S)TEM and EELS to study hydrogenation of individual Pd nanoparticles and found that the gradual increase of hydrogen concentration during the formation of β-phase Pd hydride was occurred in nanoparticles below 15 nm and that the hydrogenation pressure became lower with decreasing particle size.^22^ Therefore, there is opportunity to continue to explore this system; for example, by investigating both oxidation and hydrogenation in the same measurement conditions, and furthermore with interchangeable capability.

Here, we investigate the phase transition of Pd in both oxidizing and reducing environments. ETEM allows us to study, for the first time, sequential exposure of Pd to O_2_ and H_2_ in the same TEM conditions. First, our results confirm that, under TEM observation, oxidation occurs at step edges but we also show that it can occur at terraces. Second, and perhaps the most important result, we observed a novel process where previous exposure to H_2_ suppresses new oxidation of the Pd surface. Third, we show by EELS that this process, suppression of oxidation by previous exposure to H_2_, is not due to the formation of bulk β-phase Pd hydride. And we also demonstrate that this process is not present in Pt. Finally, we discuss the hypothesis to explain this phenomenon: formation of surface–Pd–hydride suppresses the new oxidation. Observation of this suppression process may lead to new interesting discoveries about this important metal–gas interface.

## Experimental

For ETEM study, we prepared wedge-shape Pd by electrochemical polishing and argon ion milling of Pd wires (99.95% and 0.2 mm of diameter). For the electrochemical polishing, we used Au-ring electrode as an anode and mixed solution of hydrochloric acid and ethanol. After the electrochemical polishing, we heated the Pd wires at 423 K to remove any remaining solution, and then, we further polished the Pd wires by argon ion milling. For comparison, we also studied wedge-shaped platinum which was prepared by electrochemical polishing and argon ion milling of Pt wires (99.95% and 0.25 mm of diameter). For the electrochemical polishing of Pt wires, we used mixed solution of CaCl_2_, H_2_O, and acetone. The ETEM (Titan G2 ETEM) is equipped with a corrector for the spherical aberration of the objective lens and an EELS spectrometer (Gatan GIF Tridiem 863). The acceleration voltage was 300 kV. The specimens were studied at room temperature under O_2_ and H_2_. We recorded images using an electron current density of 10 A cm^−2^ by Thermo-Fisher Ceta 16M CMOS camera with a temporal resolution of 0.5 s and a frame resolution of 4096 pixels. The Pd samples were observed with an electron beam parallel to the [110] zone axis of crystalline fcc Pd. We also performed TEM–EELS measurements under H_2_ by the ETEM operated at 300 kV without a monochromator at a dispersion of 0.02 eV per channel. Five samples were studied and the results were consistent across.

## Results and discussions

**Fig. 1:**
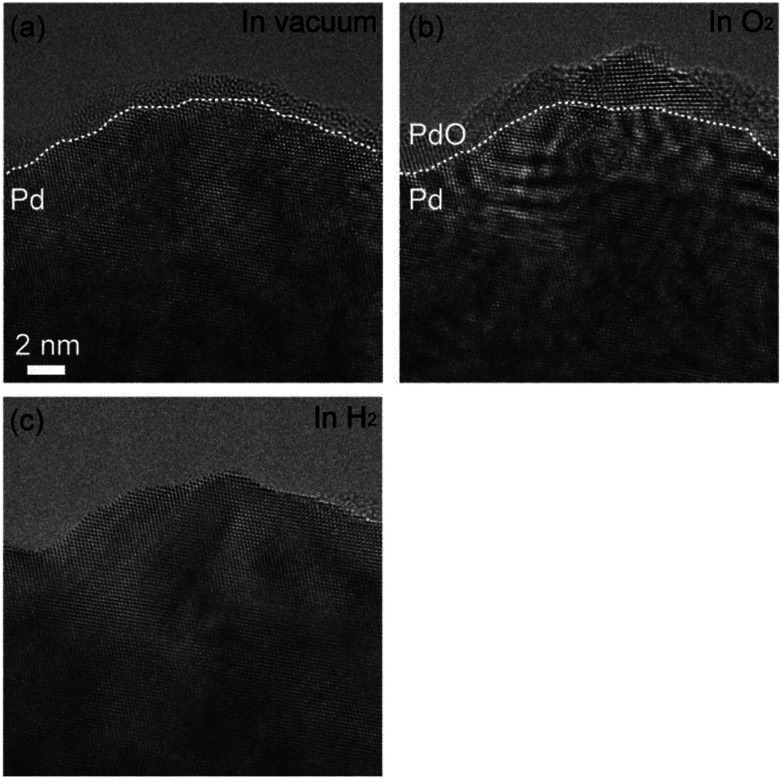
Cleaning of an as-prepared Pd surface. (a) An ETEM image of as-prepared Pd foil. Hydrocarbon contamination covered the Pd surface. (b) The surface of the Pd foil in O_2_ (100 Pa) after elimination of hydrocarbon contamination by intensive electron irradiation: PdO film was formed on the Pd surface. (c) The surface of the Pd foil in H_2_ (100 Pa): the PdO film was reduced to metallic Pd.

Fig. 1(a) shows as-prepared Pd foil. The surface of Pd foils was covered by hydrocarbon contamination. The contamination was eliminated by intensive electron irradiation in O_2_. After the elimination, the Pd surface was covered by crystalline material of several nanometers (Fig. 1(b)). Measurement of the distances between crystal planes showed that the crystalline material was Pd monoxide.^18^ After exhaustion of O_2_, the PdO film still remained, but after introduction of H_2_, the PdO was immediately and completely reduced to Pd (Fig. 1(c)). EELS measurement showed that there was no bulk Pd hydride.

**Fig. 2:**
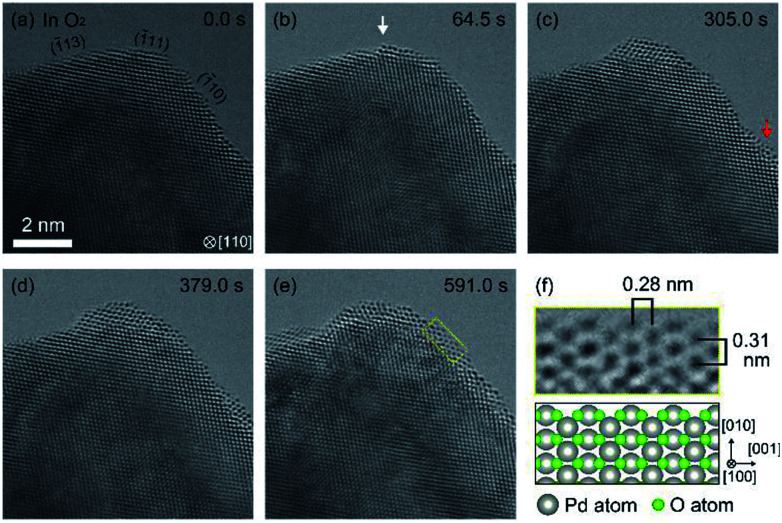
Oxidation processes of the Pd surface in O_2_ after 10 minutes exposure to H_2_ (Fig. 1(c)). (a) shows the sample just after starting observation. (b) shows an instance, after 64.5 s of oxygen exposure, where the oxidation started at a step edge (white arrow). (c) shows an instance where the oxidation started at a terrace (red arrow). The oxidation proceeded on the Pd surface as shown in (d), and finally, the Pd surface was completely covered by PdO after 591.0 s. (f) shows enlarged ETEM image of yellow rectangle region in (e) and schematic illustration of PdO (tetragonal, *a* = 3.051 Å, *c* = 5.495 Å).^18^ The gray and green balls in the schematic illustration represent Pd atoms and O atoms, respectively.

After the PdO film was reduced, we exhausted H_2_ and then introduced O_2_. The duration of H_2_ exposure was about 10 minutes. After exposure to O_2_ in 305 seconds, we observed oxidation happening in both step edges and terraces (Fig. 2(a)–(c)). Then, the oxidation proceeded on the Pd surface (Fig. 2(d)), and finally, the Pd surface was completely covered by the PdO film (Fig. 2(e)). It should be mentioned that electron beam induced the oxidation, as demonstrated by a previous study.^21^ It should also be pointed out that TEM images are projections, and therefore, the geometry of the sample perpendicular to (or behind) the electron beam is unknown. Therefore, there is inherent limitation to knowing the geometry of the area where the oxidation started. For example, the area we viewed as a terrace could have been a step edge in the direction perpendicular to the beam. Nonetheless, as more data becomes available for this system then this conclusion could become stronger.

**Fig. 3:**
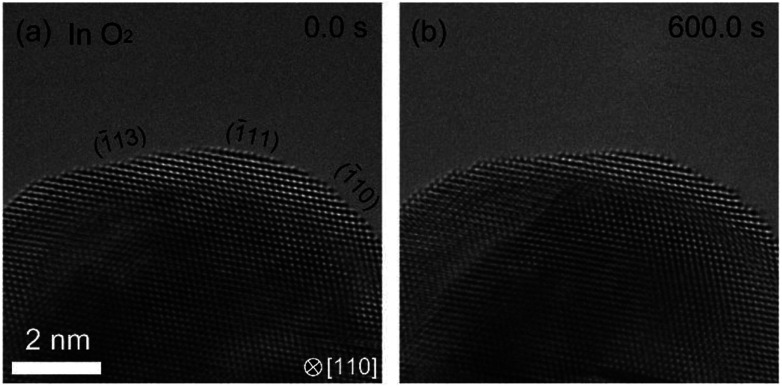
Dependence of history of hydrogen exposure on the oxidation of palladium. After the sample was kept under H_2_ in 90 minutes, we observed the Pd surface in O_2_. (a) shows the Pd surface just after starting observation. (b) shows that the Pd surface is not oxidized in 600 s where the surface of Pd is completely covered by PdO after the short exposure to H_2_ (Fig. 2).

In the hydrogenation–oxidation sequence, we also studied the effect of the duration of the H_2_ exposure. We found that there is a dependence of hydrogen exposure on the oxidation of Pd. After short H_2_ exposure (10 minutes), Pd can be oxidized as shown in Fig. 2. However, after long exposure (over 90 minutes), the oxidation of the Pd surface was not possible (Fig. 3). To our knowledge, suppression of oxidation by prolonged exposure to H_2_ has never been reported.

**Fig. 4:**
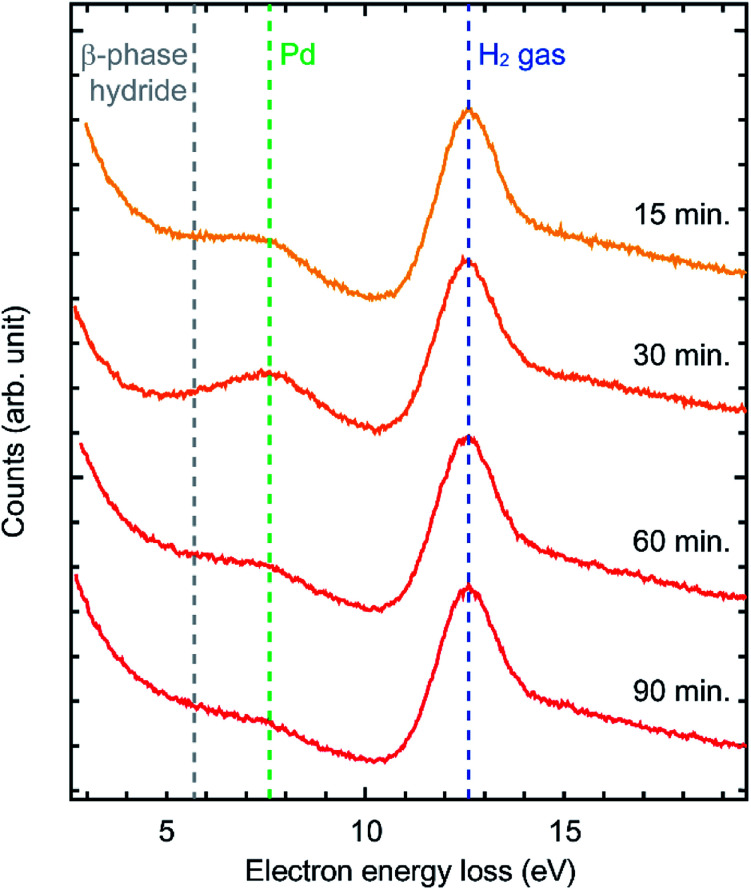
EELS evidence that bulk β-phase hydride is not formed. EEL spectra were acquired 15, 30, 60 and 90 minutes after H_2_ pressure reached 100 Pa. Two peaks were observed at 7.7 and 12.6 eV which were derived from Pd and H_2_ gas, respectively. A shift of the Pd peak from 7.7 eV to 5.7 eV which is derived from bulk β-phase hydride was not observed. This result showed that Pd was not bulk hydrogenated by 90 minutes exposure to H_2_.

Our initial hypothesis to explain this phenomenon was that the bulk Pd was hydrogenated into bulk β-phase hydride which then suppressed further oxidation. However, in the literature, at room temperature, hydrogenation of Pd requires much higher H_2_ pressure than that in our experiments.^22^ In order to verify bulk β-phase hydride, we performed EELS measurements (Fig. 4). EEL spectra were acquired from the area of about 50 nm in diameter including the surface of Pd sample which is selected by a spectrometer entrance aperture. In the spectra, two peaks were observed at 7.7 eV and 12.6 eV. Previous studies showed that the peak at 7.7 eV is derived from Pd and the peak at 12.6 eV is derived from H_2_ gas.^22^ As for the Pd peak, previous studies showed that when β-phase hydride is formed, the peak at 7.7 eV shifts to about 5.7 eV.^22^ However, in our data, the position of the Pd peak was not shifted even after 90 minutes. This suggests that the formation of bulk β-phase hydride did not occur. Therefore, the formation of bulk β-phase hydride is not the mechanism for the suppression of the oxidation.

**Fig. 5:**
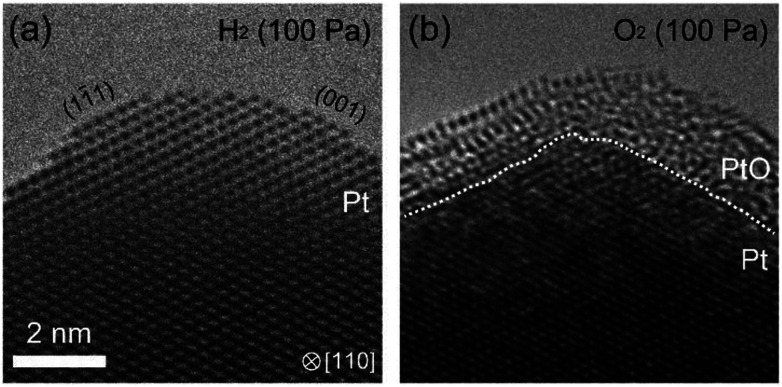
Oxidation of the surface of a wedge-shaped platinum after 90 minutes exposure to H_2_. (a) shows the platinum surface in H_2_: the platinum surface was enclosed by Pt{001} and Pt{111}. After the platinum sample was kept in H_2_ in 90 minutes, H_2_ was exhausted, and then, O_2_ was introduced. As shown in (b), in O_2_, the platinum surface was covered by amorphous platinum oxide with a thickness of several nanometers. This result shows that the suppression of oxidation is not occurred in Pt.

Another interesting question was to see the suppression of oxidation by prolonged exposure to H_2_ existed in other metals. To answer this question, we studied platinum using the same ETEM conditions and protocol (reduction–oxidation sequences). Fig. 5 shows the ETEM results. In H_2_, metallic Pt was observed (Fig. 5(a)). In O_2_, after 90 minutes exposure to H_2_, the Pt surface was covered by amorphous Pt oxide (Fig. 5(b)) as previously seen in ref. 23. This result shows that the oxidation of Pt was not suppressed even after 90 minutes exposure to H_2_. Therefore, it is clear that H_2_ is interacting with Pd in a process that is suppressing subsequent oxidation, and that this process is not existent in Pt. This is very important because it is well known in the literature that hydrogen atoms penetrate palladium but not platinum because the heat of solution of hydrogen in platinum is very large.^24^ And this is remarkably consistent with our observations of suppression in Pd but not in Pt.

As mentioned before, the formation of bulk β-phase hydride is not the process by which the oxidation was suppressed. However, surface hydride, as a barrier that prevent further oxidation, is possible. And this corresponds to our hypothesis on the process responsible. However, hydrogenation limited to the surface of Pd cannot be observed neither by ETEM nor EELS. It is possible that the dilute bulk α-phase hydride exists in addition to surface hydride. It is very likely that interstitial hydrogen atoms in the bulk α-phase hydride could diffuse to the surface and/or subsurface and contribute to suppress the oxidation of the Pd surface in O_2_. However, confirmation of our hypothesis is outside the scope of our measurement methods and this work.

We can however look to the literature for information about the surface hydrogenation of Pd. Adsorption of H atoms at the subsurface of Pd has been studied by means of temperature programmed desorption (TPD),^25^ low energy electron diffraction (LEED),^25^ and other methods.^26^ These studies showed that adsorption of H atoms on the subsurface is possible and depends on the crystal plane, coverages of adsorbed H_2_ molecules, and temperature.^25^ Conrad *et al.*^25^ showed that even at low pressures (<1 Pa), the work function of the Pd(110) and Pd(111) surfaces increases due to the negative charge^27,28^ of the adsorbed H atoms. They showed that the adsorption of hydrogen can occur at room temperature and at very low pressures. Annealing sequence measurements also showed that some hydrogen penetrates the bulk, and that after short times the hydrogen can come back to the surface as adsorbed atoms.^25^ In these conditions, bulk diffusion calculations yield an average penetration distance of about 200 microns in 1000 s of exposure.^25,29^ Further proof of the subsurface occupation of hydrogen atoms was given by Behm *et al.* who showed that on Pd (110), subsurface occupation with coverages larger than 1 monolayer can occur even at lower temperatures (170 K).^30^ Similarly, neutron scattering measurements showed that even at very low pressures hydrogen can occupy subsurface sites in Pd.^31^ Therefore, our hypothesis is consistent with the literature: our experimental conditions in the ETEM have a hydrogen pressure (100 Pa) that is high enough to cause surface adsorption and subsurface occupation of hydrogen atoms.

## Conclusions

The observation of suppression of oxidation by prolonged exposure to H_2_ raises very interesting questions about the gas–metal interface in Pd under O_2_ and H_2_. This process is further made more interesting by the absence of bulk β-phase hydride and the absence of this phenomenon in Pt under the same experimental conditions. This observation could eventually lead to the discovery of new surface process involving the surface hydrogenation of Pd. Though in this work, we focused on the discovery, systematic experiments for understanding conditions are needed.

## Conflicts of interest

There are no conflicts to declare.

## Supplementary Material
